# Gender Equality and Social Inclusion in Relation to Water, Sanitation and Hygiene in the Oromia Region of Ethiopia

**DOI:** 10.3390/ijerph18084281

**Published:** 2021-04-17

**Authors:** Geteneh Moges Assefa, Samiha Sherif, Jose Sluijs, Maarten Kuijpers, Tamene Chaka, Arsema Solomon, Yeshitila Hailu, Muluken Dessalegn Muluneh

**Affiliations:** 1 Monitoring, Evaluation and Research Department, Amref Health Africa in Ethiopia, P.O. Box 20855, Addis Ababa 1000, Ethiopia; mulusef@yahoo.com; 2 Freelance Consultant, San Jose, CA 95129, USA; ssherif004@gmail.com; 3 Amref Flying Doctors, Leiden, Schuttersveld 9, 2316 XG Leiden, The Netherlands; Jose.Sluijs@amref.nl (J.S.); Maarten.Kuijpers@amref.nl (M.K.); 4 Amref Health Africa in Ethiopia, P.O. Box 20855, Addis Ababa 1000, Ethiopia; Tamene.Chaka@amref.org (T.C.); Arsema.Solomon@amref.org (A.S.); yeshitila.hailu@Amref.org (Y.H.); 5 School of Nursing and Midwifery, Western Sydney University, Sydney 2751, Australia

**Keywords:** women, schoolgirls, social inclusion, gender, WASH service

## Abstract

The main purpose of the study was to deepen the understanding of gender and social inclusion in the context of water, sanitation, and hygiene (WASH) in the Oromia region of Ethiopia. An explorative qualitative study was conducted in three districts of the Oromia region using gender analysis frameworks. Twenty-one key informant interviews and nine focus group discussions were conducted. Findings showed 52% of households in the study area have basic service level water, 29% have basic service level sanitation, and 14% have basic service level hygiene. Women, girls, and people living with disability disproportionately experience poor access to quality WASH services. Women and girls participate in unequal domestic labor related to water management which often exposes them to discrimination and violence such as rape, abduction, and assault. Overall, women, girls, and other socially excluded groups are rarely consulted and engaged by local actors. This results in incongruent policy and political commitment which limits action at the grassroots level. Integrating gender equality and inclusion efforts into local governance agendas can help to increase access to and the quality of WASH services. These efforts must advocate for moving beyond gender parity to promote gender transformative approaches and inclusion to realize better WASH services for the communities they serve.

## 1. Introduction

Achieving inclusive growth and development is a challenge for many developing countries, including countries in sub-Saharan Africa [1,2]. Despite significant progress over the years, there remains poor access to basic infrastructure and services such as water, sanitation, and hygiene (WASH), which contributes to poor health outcomes. It is critical that sub-Saharan African countries actively mobilize resources to attain universal WASH services by 2030 to improve health outcomes and achieve Sustainable Development Goal (SDG) 6-related targets [3,4,5]. This approach should specifically target socially excluded groups (SEG), such as women and people living with disability (PLWD) who disproportionately experience poor health outcomes and risks related to WASH. Socially excluded groups are continuously underrepresented at all levels, including in household level WASH decision-making, [6,7,8] leaving most WASH decision making structures and processes to be dominated by able-bodied men. This has resulted in WASH service delivery being insensitive to the needs of women and other SEGs at both the community and societal levels. Additionally, socioeconomic factors such as disproportionate access to education, and thus low literacy levels, greatly inhibit the economic status of women and girls and limit their participation in WASH decision-making [9,10]. Socially and culturally induced gender roles disproportionately assign most WASH management activities, such as fetching, storing, and treating water to women and girls [11,12]. Female children who are generally more involved in household activities than male children are also at a higher risk of experiencing WASH related illness, such as diarrhea; however, illness is also associated with other risks factors, including the hygiene of female caretakers, household practices, and perceptions of WASH related illness (e.g., diarrhea) [13,14,15]. The reality of water fetching from long distances also exposes girls and women to crime, gender-based harassment, sexual assault, and chronic stress [16,17,18,19]. Poverty and low awareness of gender and social inclusion topics increases the vulnerability of marginalized groups and often inhibits their social mobility at the individual level [1,12,20,21].

Furthermore, inadequate infrastructure supporting sanitation and hygiene frequently pushes many women to unhealthy coping strategies. To avoid urination, many women often limit their food and water intake [22]. These negative coping strategies are associated with adverse health outcomes and psychosocial stress. For schoolgirls, the lack of adequate WASH resources and menstrual hygiene management (MHM) facilities in schools creates additional burdens. Girls reported experiencing substantial fear and embarrassment during menses because of the stigma associated with menstruation. Unsafe toilets, insufficient water access, and limited access to MHM materials are commonly associated with increased school absenteeism among girls. Studies conducted in sub-Saharan Africa suggest that poverty, inadequate MHM resources (pain management, pads, water and sanitation facilities) and limited psychosocial (self-confidence, attitude) support are key drivers for poor school attendance for girls [23,24,25,26,27].

In Ethiopia, the WASH context is starkly similar to the broader sub-Saharan African context. The Government of Ethiopia has made significant strides towards achieving universal WASH services by 2030, yet inadequate and inequitable access to WASH services remains a challenge. Ethiopia has developed a “One WASH National Program” (OWNP) to facilitate the development of one plan, one budget, and one report for the wider WASH sector with key sector ministries that have committed to improving community WASH [28]. Though the program is in implementation, availability and proper use of latrines, water, and hand washing facilities remains low in Ethiopia. The approach requires enhanced investments and amplified efforts across the WASH sector to deliver strong outcomes [29,30,31]. Similarly, Ethiopia’s 2017–2022 Growth and Transformational Plan (GTP) centers WASH, inclusion, and the needs of women, youth, and vulnerable people, however the intended results remain well behind targets. These progressive policies are welcomed and necessary but must also be followed with strong and coordinated implementation. Comparably, Ethiopia has committed to an inclusive society in alignment with the SDG’s mandate to “leave no one behind [32,33,34]” by establishing disability as a cross-cutting sector of development and uplifting the needs of PLWD through both Ethiopia’s GTP and the National Plan of Action of Persons with Disabilities (2012–2021). Nonetheless, SEG continue to experience marginalization and face limited access to WASH services and infrastructure [35]. According to a report on disability jointly issued by the World Bank and WHO [36], there are an estimated 15 million children, adults, and elderly persons with disabilities in Ethiopia, representing 17.6% of the population. About 95% of all PLWD are estimated to live in poverty and over half (55%) of PLWD in Oromia depend on family, neighbors, and friends for their living. These social determinants have clear implications for WASH access and WASH related health outcomes which should be explored and documented. Limited literature on barriers faced by SEG, such as PLWD, the elderly, and women, suggests a need for more research. By exploring the context in Oromia, Ethiopia’s most populous region, the study aims to present a qualitative analysis of WASH services and outcomes using a gender and social inclusion (GESI) lens. The goal is to better understand the experience of Oromia’s population (with a focus on SEG) related to WASH and make recommendations to improve WASH services in the region.

## 2. Materials and Methods

### 2.1. Setting, Design and Population

According to the Central Statistical Agency (CSA) of Ethiopia, the total population of Oromia Regional State is about 35.5 million, with 82% living in rural areas [37]. This accounts for 37.7% of the total population of Ethiopia. The Oromia region is located within the Ethiopian Rift Valley, whose lakes have experienced unstable water level fluctuations over the last two decades due to climate variability. The situation is expected to worsen considering the increasing imbalance between a continuously growing population and a degrading resource base. The Rift Valley Lakes Basin (RVLB) in Oromia is a hydrologically closed basin, characterized by terminal lakes (those with no surface water outlet), that has experienced over abstraction, siltation, environmental degradation, population pressure, industrialization, urbanization, infrastructure development, and pollution over the years. It contributes 15% of Ethiopia’s national water supply, yet 60% of the nation’s total population lives in the areas surrounding the RVLB.

The districts in Oromia targeted for the study are among lowland areas where most natural water sources are not suitable for drinking due to high temperatures and high fluoride content [38,39]. According to a WASH service level baseline study in Oromia in 2017, water, sanitation, and hygiene service levels are extremely low. Furthermore, the participation of women and other SEG in WASH related decision making is low, despite government bodies making efforts to facilitate forums to engage marginalized groups [40].

### 2.2. Sampling, Tools and Data Collection

To better understand the landscape of WASH services and GESI in the region, the study employed an explorative study design in three woredas of the Oromia region in Ethiopia: Arsi Negele, Shashemene, and Adamitulu Jido Kombolcha Woredas. Both rural and urban kebeles/sub-kebeles were selected from each woreda for inclusion in the study sample. The study woredas were selected randomly from West Arsi zone and the sample size was determined using the point of saturation for qualitative data analysis. Purposive sampling techniques were used to select key informants (Table A2) and focus group discussion (FGD) participants (Table A1) including representatives from key government offices, schoolgirls, and disabled persons.

The study design was informed by multiple gender analysis frameworks (GAF) including Women’s Empowerment Framework, Gender Analysis Matrix (GAM), gender planning emphasizing practical and strategic needs, Harvard Analytical Framework, and Moser Gender Planning Framework. The study intended to explore the relationship between gender and WASH in the three woredas of the Oromia region to better inform development projects. Special interest was given to gender disparities across the social, economic, religious, and political contexts of those living in the study area [39,41]. Due to the influence of gender norms in the region, the study focused on the dimensions of gendered division of labor, workload related to WASH/water management, gendered access to resources, and decision-making processes and norms based on existing policies and institutions.

Various international, national, and local documents concerning gender were reviewed and assessed prior to data collection. Twenty-one informant interviews (KII) and nine FGDs were conducted with local men and women, schoolgirls, members of WASH committees, representatives of marginalized groups (people living with HIV; disability), woreda administrators, and representatives from the woreda level health office, education office, water and irrigation office, women and children/youth office, and social affairs office. Data collection was guided by a pre-tested semi-structured interview guide and FGD guides which focused on gender and social inclusion issues. FGDs with women’s groups and schoolgirls were facilitated by female data collectors to facilitate open dialogue.

The different dimensions considered during the gender analysis are briefly presented as follows:

**Dimension 1**: Gendered division of labor and workload. In this dimension, we examined the different WASH related roles and responsibilities of women/girls and how this affects their ability to perform other day-to-day activities. This dimension also examined how political, economic, and social systems structure the gendered division of labor within households and communities.

**Dimension 2**: Access and control over resources, and household and community level decision-making. This dimension examined the power of girls, women, the elderly, and other SEG and their control over and access to resources at the household and community levels.

Additionally, the benefits of control over resources and the types of resources that girls, women, and other SEG can access were explored. The effects of (gender-based) inhibited access to resources was also analyzed in phase two of the study.

**Dimension 3**: Girls, women, and other SEG’s sense of self-efficacy and ability to partake in decision-making. This dimension mainly focused on women, girls, and other SEG’s decision-making power related to awareness of and ability to use WASH services. It also included strategies to ensure their right to access WASH services in the community.

**Dimension 4**: Laws, policies, and institutions. Different legal rights affect the capacity of each gender to access services or resources and make decisions based on existing policies and institutions. In this gender analysis, we reviewed national and local documents with respect to gender. Based on the above dimensions, we analyzed factors affecting gender relations in inclusive WASH services.

### 2.3. Analysis

All qualitative interviews were audio-recorded, transcribed, translated, and analyzed using ATLAS Ti. Content summaries of the interview transcripts were created around major codes and sub-codes. The resulting matrices were used to explore themes across informants to identify common issues related to the study variables.

### 2.4. Term Definitions

The WHO/UNICEF Joint Monitoring Programme for Water Supply, Sanitation and Hygiene (JMP) uses.

#### 2.4.1. Safely Managed Service Levels

Water: drinking water from an improved water source, which is located on premises, available when needed, and free from fecal and priority chemical contamination.

Sanitation: use of improved facilities which are not shared with other households and where excreta are safely disposed in situ or transported and treated off-site.

#### 2.4.2. Basic Service Levels

Water: drinking water from an improved source, provided collection time is not more than 30 min roundtrip, including queuing.

Sanitation: use of improved sanitation facilities which are not shared with other households.

Hygiene: availability of handwashing facilities on the premises with soap and water.

#### 2.4.3. Limited

Water: drinking water from improved sources for which collection time exceeds 30 min roundtrip, including queuing.

Sanitation: use of improved facilities shared between two or more households.

Hygiene: availability of handwashing facilities on premises without soap and water.

### 2.5. Ethical Considerations

Research participants were not required to be involved without their will and were notified of their right to withdraw at any time during the study. Participants were assured that withdrawal from the study at any time did not have any consequences. Additionally, participants were informed of the purpose of the study, the methods, estimated time commitment, selection process, how the results will be used, and the intended impact of the study before their informed consent was collected. The confidentiality of the data was guaranteed by preserving the anonymity of the study participants. Identifying information (names and addresses of respondents) was not included in the data collection instrument. Letters of support or approval to conduct fieldwork were obtained from the respective Bureaus of Health, Zonal Health Departments, and Woreda Health Offices. The officials were briefed on the proposed assessment, the purpose, methodologies, ethical issues, type of activity, level of human activity, and the expected duration of data collection.

## 3. Results

Nine focus group discussions with an average of nine participants in each group were conducted. One group was composed of people living with HIV (PLHIV) from both sexes, one group exclusively with women, one group exclusively with men, two groups of elderly (both sexes), and three groups of schoolgirls. Twenty-one key informant interviews (13 women and 8 men) were conducted with individuals from various backgrounds. Key informants included PLHIV, PLWD, health extension workers, woreda administrators, and representatives from woreda level offices including education, water, social affairs, women and children, and health.

### 3.1. Water

Water unavailability or inadequacy was the most common problem raised by the study participants. Over 52% (6% safely managed, 46% basic) of households in the study area have a basic service level of water, which means the water is coming from an improved source and collecting the water takes less than 30 min [40]. However, many residents who have access to potable water points face repeated interruption to the service. In many lowland areas, the water is not suitable for drinking because of high water temperatures and the presence of fluoride, which is not recommended for drinking purposes. Nonetheless, study participants often consume this unsafe water when they are unable to collect from potable water sources. A woman FGD participant from Adamitulu spoke to the the importance of prioritizing water by stating, “before we talk about hygiene, it is better to talk about drinking [water].”

In terms of geographic water distribution, kebeles (the smallest administrative structure in Ethiopia) in the highlands have greater access to spring water than those in the semi-highlands, and those living in the lowlands experience major challenges to hole-water access. According to a KI from the Shashemene Water Office, “out of the 22 kebeles located in the lowlands, 11 kebeles (50%) have a critical water shortage.” Deep-hole water is available in the lowlands; however, it is unsafe for consumption due to high fluoride content. Another barrier to water access in the lowlands is the resource intensive nature and difficulty of extending water from the highlands and semi-highlands to the lowland kebeles. Because of the long travel distance to fetch water and irregular availability of potable water, many are left with few options but to use non-potable water sources. Almost all FGD participants reported that it is not uncommon to have no access to potable water for more than a week. In some areas, people even resort to breaking the water pipeline and diverting the water for their use. This is usually done to avoid travelling long distances to the water point, in which often times water is not available by the time they arrive to the water point. Some even opt to use hot surface water for washing/hygiene purposes, despite associated health risks.

Most study participants reported that they often travel up to seven kilometers in search of water. When they travel long distances, people commonly use carts (Gari in the local language). In other cases, women carry the water by hand or on back, use donkeys, or they may pay for daily laborers if they can afford to. If they prefer to hire a Bajaj or Gari, they may pay about 0.4 USD per Bajaj or 0.3 USD per cart to transport a 20 L jerrycan. In many cases, women wake up early in the morning (as early as 5:00 am) and travel up to 8–10 h roundtrip, including long queue times.

#### 3.1.1. Gender and Water

The study findings suggest that women have greater responsibility in collecting, handling, storing, and treating water in the study areas. Except in some places where husbands help to collect water with the help of children, women take on the primary responsibility. Focus group participants expressed that women are regularly assigned WASH related responsibilities due to local gender and societal norms.

“*I don’t know the reason but the burden of all activities regarding water lies on women. Sometimes men get angry if we could not wash their clothes due to other workload. They do not care about activities at home. This is due to societal influence*.”

(Women FGD participant from Adamitulu)

Sometimes husbands may help their wives by using either donkeys or carts to collect water from very far places. The contribution of husbands in a few localities is more pronounced when their wives are sick, pregnant, or are at the early postnatal period. This is dependent on their awareness of gender equality and their commitment to collecting water for household consumption. Most often, however, women and girls shoulder the labor of water management.

“*We hear about gender equality and the rights of women. Nevertheless, no one is helping us to make injera or cook. We are engaged in duties both inside and outside of the home. Girls keep livestock after school. Therefore, I cannot say the right of females is respected. There is a heavy burden on us. Males are not helping us by taking responsibility in the home. We [women] do all activities like cooking, sanitation, hygiene and livestock rearing. I do not see [anything] about gender equality*.”

(Women FGD participant from Adamitulu)

With regards to water consumption, male FGD participants expressed that females generally consume more water, mostly associated with “…*staying for longer periods of time at home, preparing food, washing clothes, caring for child hygiene, making major decisions regarding use of household water and we [men] support them by bringing water treatment materials*” (Male FGD group participant from Arsi Negele)

When asked about water management related challenges their husbands faced, women FGD participants shared that males do not save water and that they expect that their wives are fully responsible for ensuring adequate water is available for their household.

“*Males also consume huge quantities of water. They don’t save water and even they don’t want to hear advice from us about water saving. One man may consume more than a Jerrycan for bathing only, ahh they do not save and consume the water collected by females. Surprisingly, if we couldn’t make it ready, they may get disappointed or angry to the extent they may beat us*.”

(Women FGD participant from Adamitulu)

With regards to the efforts made by the local government, a KI from the Shashemene Woreda Water Office said that women, including girls up to age seven and the elderly, are at risk of injury when fetching water using 20/25 L jerrycans. The water office had a series of consultative meetings with the Woreda Gender and Child Affairs Office to inform communities of the risk of injury and poor health outcomes, and to advise fetching smaller loads by providing 10 L jerrycans to the community.

Although women are primarily responsible for water collection and management activities, they are not usually consulted during the design and construction of water points. This often results in negative consequences, including failures related to site selection and water points insensitive to their needs. A KI from Shashemene Woreda Water Office shared that the lack of community participation during design and questionable functionality of the water points has even resulted in legal action in some areas:

“*There are public drinking water standpoints that were constructed by NGOs. The NGOs themselves and the contractors decided the design and sites of construction. There are many non-functioning public drinking water standpoints in this woreda due to their design and inappropriate site of construction. Some of them have no water and others are not accessible to the communities, especially for children and disabled persons. For that matter, some people are under [legal] investigation related to these problems*.”

At the kebele level, a majority of the villages where there are public water points assign women to manage the money collected for water services. According to the KI from Shashemene Woreda Women and Children’s Affairs Office, “*women are more trusted to manage money collected at the community level. Many WASH committees have problems related to missing or abusing money collected, particularly by men. So, to solve such problems, currently, women are highly engaged in administration and management of the collected money. So they can use that money for maintenance of the water point*”.

#### 3.1.2. Social Exclusion

The elderly and PLWD are often overlooked in regard to accessible WASH-related services. As a result, they end up fetching water from nearby, unprotected or unsafe water sources since it is easier than travelling long distances to water points. Shortening the distance between villages and water points alone, however, will not solve the problem for SEG unless the design of public drinking water points is inclusive. Currently, officials have expressed that women and other SEG are almost never consulted or involved in the design and construction of water points.

“*There are no local policies, strategies, programs or frameworks in place to promote inclusion of SEG, including women, in decision making on WASH related development activities. To the best of my knowledge, I have never seen any water or sanitation point designed appropriately for easy access for persons with disabilities*.”

(KII PLWD Representative from Arsi negele)

“*We do monitoring of WASH-related activities and their progress. The problem is that we do not monitor its [social] inclusiveness, especially for PLWD. We mainly focus on providing water for the community as much as possible. The issues of inclusiveness for PLWD, women, children, elderly, etc. are almost less understood or overlooked*.”

(KII from Shashemene Woreda Water Office)

This finding also shows that SEG are not only PLWD, elderly, and children. It also includes people who have limited resources, such as donkeys or carts, and therefore do not have equal access to water due to the long distances travelled to fetch water. This implies that in environments hard hit by critical water shortage, the poor, PLWD, elderly, and children suffer disproportionately.

The study also found that in addition to poor advocacy and design related exclusion, PLWD are also discriminated against in many ways by the community and excluded from social activities. One example shared by a key informant is discrimination related to marriage customs and events.

“…*Communities don’t believe PLWD are capable and important in development programs, maybe they believe we are sin. For example, in our culture we do not even send people with disability for jarusma (it’s an event where elderly people are sent to ask a woman family for marriage) as her family will consider it as disrespectful and unacceptable*…”

(FGD PLWD from Arsi negele)

#### 3.1.3. Implications of the Water Situation

The findings suggest that women, children (specifically girls), PLWD, and PLHIV are more affected by the implications of water access compared to men and boys who have limited roles and responsibilities related to water collection, handling, and use, particularly in the lowlands. The time commitment necessary to search for water means women spend most of their time away from home, often having to leave their children unattended.

“*If we take the condition of our village, not only me, but a majority of females wake up at 6:00 am and go searching for water. We also spend our time waiting for our turn [queue] and travel. We arrive home after 4:00 pm. By then our children could be sick due to hunger [untimely care], girls and we (Women) are also exposed to gender based violence, and our husbands are not understanding about what we face on the way. This brings an additional burden to our lives*.”

(An FGD participant from Adamitulu)

A KI from Shashemene woreda also mentioned that girls and women frequently encounter violence such as rape, abduction, and other psychological and physical assaults while travelling long distances in search of water. Children commonly ride donkey carts to fetch water and carry water beyond their capacity. As a result, many children become injured because of physical incapacity to manage the loads. It is also common for water to be unavailable by the time women make it to the front of the queue. This unreliability of securing water from water points results in households often relying on stored water that may be contaminated (resulting in illness).

”…*it is not only the children that get hungry; women also stay for long hours because of the long time wasted in search of water. They may stay without eating even breakfast since they are leaving in the early morning before cooking food. Due to the irregular availability of water, families are forced to consume water stored for a long time using tankers [large buckets, might be produced worms]. As a result, health problems like diarrhoea, especially under five diarrhoeas are very common in the lowland kebeles*.”

(KI from Shashemene Woreda)

#### 3.1.4. Implications for School Attendance

Only 37% of the schools in the survey area have basic water service and many schools experience high frequency of interruptions [40]. Critical shortage of water affects both the health and the hygiene of people, including the menstrual hygiene of schoolgirls. According to FGD participants, there have been many incidents where teachers prevented girls from attending classes due to poor personal hygiene. Additionally, many school children frequently miss classes due to the demands of travelling long distances searching for water for their households.

“*Going too far to fetch water creates a problem in our school [attendance]. One might be coming late without any preparation for school because the “first thing is life” before school. Even after school, we go far distant in search of water and we will not have time to study*.”

(FGD Schoolgirls in Adamitulu)

“*I wake up in the morning at 5:00 local time to fetch water. If my class is during the morning shift and there are too many queues, I miss class because I have to wait for water. If I’m in the afternoon class, I may go to school without doing homework and assignments*.”

(FGD Schoolgirls in Shashemene)

“…*we do a lot of activities including the collection of firewood, sometimes males follow us to request umm (sexual intercourse). If there are not sanitation tools like water and soap available, we may have body odour and we get ashamed to go to school during the weekdays. This affects our school attendance*.”

(FGD Schoolgirls in Arsi Negele)

#### 3.1.5. Economic Implications

Inadequate access to water both increases the burden of women’s daily activities and affects their participation in other economic activities. After travelling long distances in search of water, most women return home to several domestic demands. As a result, women lack the time to engage in income-generating activities outside of the home.

“…*Because we (women) stay away for long, our livestock like goats and calves may get lost. Therefore, this affects our economic status. We also lack time for cooking and so we do it in the night. We have no time for breaks and this affects our wellness and health*.”

(A female KI from Arsi Negele social affairs)

### 3.2. Sanitation

Households in the study area typically use locally available materials to construct toilets. The toilets usually have poor durability and fail to stand for long periods of time (erosion damages the toilets during the rainy season). In some areas, the soil texture is not suitable for the construction of durable toilets. This can result in flooded soil, which is usually covered by sand to halt the flooding. To avoid flooding, many households who experience high flooding during the rainy seasons no longer construct toilets. Other options are few, and often unsafe and not girl-friendly, leading many women to avoid latrine use which may result in health complications.

Of the study participants included, only 23% (2% safely managed and 21% basic) have basic or safely managed sanitation service levels within their households. The rest use limited sanitation facilities (6%), unimproved sanitation facilities (46%), and open defecation (25%) [40]. There is a critical lack of sufficient toilets and sanitation supplies. Due to critical water shortages, there are hygiene and sanitation related issues like diarrhoea in the study woredas, particularly for lowland kebeles. Although the local government has been trying to increase latrine coverage across all kebeles, it has been difficult to meet this objective due to the poor quality of materials used for construction and the nature of the soil structure.

“*We have no sanitation facilities in our surrounding. We used to get advice from different organizations including the government to construct toilets. However, our land is not suitable. It fails within a short period [not durable], because of soil nature. It should not be wood, but rather cement is needed to build durable toilets*.”

(Woman FGD participant from Adamitulu)

There are no communal latrines in the study areas, which means people engage in open defecation when there is no toilet available in their household. FGD participants shared that, *“…usually we are going far to use open spaces which is a risk for our safety, and whenever we partake in open defecation in the daytime, it creates an itching problem to our reproductive organs. So, sometimes we want to stay until night, usually by eating and drinking less*” (Woman FGD participant from Adamitulu).

A male participant from the men’s group also supported this idea by stating “…*I guess we have a bit more privacy/freedom to use open space defecation than women, but still we are affected by different diseases like diarrhea and allergies*.” (Men FGD participant from Arsi Negele)

People also lack access to sanitation products or supplies such as soap and water, and there are no handwashing facilities near the toilets. Women tend to use tissue paper due to a shortage of water, however more often than not, their buying capacity for sanitation products is low. Sanitation is not only a problem for rural areas but also for urban areas. The study found an association between critical shortage of water and serious poor sanitation practices. According to a KI from Shashemene Woreda Water Office, there are many sanitation related problems like diarrhea and trachoma even in the urban area of Shashemene town, mainly because of water shortage.

Recently, there have been encouraging increases in latrine coverage because of the health extension program and the active mobilization of agents within the women development army (WDAs). The majority of households are believed to have individual latrines, irrespective of quality and cleanliness. However, many households usually do not use the latrines due to poor hygiene behaviors and lack of water. In areas where there are communal latrines constructed through community participation, a lack of adequate water often prohibits the delivery of necessary sanitation services (e.g., hand washing, disposal).

According to a KI from Shashemene Woreda Women and Children’s Affair Office, the attention given to the development of latrines in rural areas by the government and implementers is very poor. Schools, religious places, and public institutions, like the court, are prioritized over the community. In other cases, the issue of latrines gets greater attention when there are epidemics of diarrhea.

“*Frequent campaigns and home visit activities take place during the epidemic periods but once the epidemics are controlled, no one raises the issues of latrine, sanitation and hygiene*.”

(KI from Shashemene Woreda Women and Children Affairs Office)

Some of the sanitation-related problems in visited schools includes a critical shortage of water, lack of sanitation products, and poor management (cleanliness and hygiene) of toilets. According to the FGD participants from Adamitulu at Karbi elementary school,

“*Though there are latrines, its management is very poor due to poor utilization. In some cases, this is because of low levels of awareness of lower grade students like below grade 5 on proper utilization of the toilets. Such children often defecate just outside the toilets as the seats are not safe for them [wider openings] and there are no handwashing facilities in schools*.”

(An FGD participant Schoolgirl in Adamitulu)

There are latrines separated for boys (four) and girls (four) but there is an imbalance between the number of rooms and the number of students. Students face long waiting times to use the toilet. Because of the queue or waiting time, students are sometimes forced to resort to open defecation.

“*No one is cleaning the toilets. I never see someone cleaning our toilets. There were four toilets for female [students] but currently only one is functional. The hole of the toilet [seat] is totally covered by feces. We cannot even get into it, even if we want to use it by cleaning, we can’t find water and handwashing facilities*.”

(An FGD participant Schoolgirl in Shashemene)

#### 3.2.1. Gender and Sanitation

According to study participants, in some places (like in Shashemene Woreda), the participation of men in the construction of latrines is low. Lack of education, attitude, and socially induced gender roles (men commonly consider it a woman’s job) are some drivers of households not building toilets. During home visits, HEWs usually pressure women to lead in the construction of household latrines which usually results in women feeling shamed into digging a hole and covering it with any available material. These latrines are often poorly constructed and lack the adequate depth needed to avoid filling too quickly. When the latrines fill, households once again resort to open defecation rather than digging again. The local belief in many villages is that it is shameful for women to openly defecate during the daytime, which is possibly why more women are committed to constructing toilets than men.

Unlike the information from Shashemene Woreda Water Office, in Adamitulu Jido Kombolcha Woreda, men do most of the construction activities. Women may help in clearing and plastering by mud, while the men dig the holes; however, the hygiene of children still remains the responsibility of women. When asked how women manage disposing of their children’s excreta, they said they take the excreta away from home and wait until it dries, then burn it. This is a common practice in the communities of Adamitulu Jido Kombolcha Woreda. According to FGD participants from Adamitulu, “*when husband’s see the feces of their children, they call their wives rather than managing it. They (husbands) feel they are not responsible for hygiene related activities at home*.” In general, the sanitation situation of the overall family lies on the shoulders of females and girls.

According to HEW KIs, women more actively participate in sanitation and handwashing-related activities at home, compared to their husbands. This is partly due to community mobilization led by WDAs in their villages. To create open defecation free (ODF) villages, HEWs coordinate WDAs to implement the sanitation aspect of the health extension package. Women are encouraged to take initiative and to urge their husbands to build and use toilets.

#### 3.2.2. Social Exclusion

Communities are strategically encouraged to construct toilets using locally available materials. However, the design and type of toilets that are constructed at the individual level are frequently weak and not accessible for PLWD and sometimes even children, women, and the elderly.

“*There is continuous auditing [monitoring] to make sure that households have constructed their toilet. The problem is that the auditing mainly focuses only on availability [of toilets] but not their inclusiveness for people who need special support*.”

(KI from Shashemene Woreda Women and Children’s Affairs Office)

“*There are no people with disability in our school; however, facilities are not friendly for them if they come to our school. The hole of the toilet is wide and children cannot use it easily. They may fall into the hole if they try to use it*.”

(Schoolgirl from Adamitulu)

Socially excluded groups are also poorly involved in the design and planning of large-scale WASH projects, despite being considered “key informant groups”. Even though socially excluded people are invited to join meetings, their attendance is usually very low, and they frequently feel they are not listened to.

“*People (both in rural and urban) are not fully aware of inclusion for children, the elderly, and PLWD. Usually socially excluded groups don’t come for meetings. It is probable they feel we (socially excluded groups) are not valued in the meetings*.”

(KII from Shashemene Woreda Women and Children Affairs Office)

#### 3.2.3. Implications of Sanitation Situation

The lack of functional household level toilets has multiple negative implications in the lives of PLWD, women, and girls. When latrines are not available, many women resort to open defecation. The study found that women are fearful of defecating during the daytime because of a lack of privacy when there is high foot traffic during the day. In such cases, women wait until it gets dark before relieving themselves which often causes strains on their health. Rampant gender-based violence, including sexual assault, also complicates open defecation for women during the night. In areas where there are latrines, the toilets are frequently not friendly for PLWD, which forces them to go to far places looking for open spaces. In some villages, poor sanitation standards cause toilets to become breeding places for flies. This creates fertile ground for communicable diseases which disproportionately affects vulnerable groups. In schools, the absence of proper toilets means students either go to the forest to defecate or wait until they go home.

### 3.3. Hygiene

Only 14% of households in the study area have basic hygiene service levels and 8% have limited hygiene service. The remaining 78% of households do not have a household level facility for hand washing. This reported finding slightly higher as compared to EDHS 2016 [40,42]. The hygiene situation of the communities was impacted by critical shortages of water, handwashing facilities, and toilets, with a disproportionate negative impact for women and girls. A FGD participant explained that poor access to water is the primary driving factor of poor hygiene practices in Adamitulu woreda.

“*If we get bathroom and water, it is easy to manage our personal hygiene. The most important thing is to have water, not the shower facilities*.”

(FGD Woman in Adamitulu)

The critical shortage of water has a direct and significant impact on the frequency of hygiene practices among women. For instance, in villages that were visited during data collection, FGD participants stated that unless a woman is menstruating, it is common to take a bath just once a year (partly attributed to lack of awareness). More commonly, women and men in many villages have access to bathing facilities anywhere between every five to nine weeks. The limited access to sanitation facilities and water means maintaining proper menstrual hygiene is difficult for women and girls, both in- and out- of school. It is especially difficult for schoolgirls who experience challenges changing their sanitary pads and panties timely due to a lack of privacy in schools.

“*We have a toilet at school but we do not go there to change sanitary pads as it is not clean for use. We also fear [feel ashamed of] taking sanitary pads from our teacher. So, if our menstruation comes at school, we may go home by interrupting our class*.”

(FGD participant from a Schoolgirl at Arsi Negele Woreda)

According to the schoolgirl FGD participants, traditionally there is a belief that if women do not keep up their menstrual hygiene by changing sanitary pads timely, they may be exposed to a disease locally known as “Michi” which can affect the reproductive organs or cause infection.

“*During the menstrual time, we get ashamed to go to the toilet in the absence of water. Because of this, we are afraid that unhygienic conditions (lack of washing) may create an abdominal problem. Traditionally, this type of disease is called “Michi*.”

(School Girl FGD participant from Adamitulu)

Often times, if schoolgirls do not have the means to maintain their menstrual hygiene, they may resort to disengaging by sitting at the back of the classroom or skipping classes until they are finished with their menstrual period.

“*Some girls may use jacket or coat from their male friends and cover their blood spot if it appears on their dress. Others may use the calendar method to make materials like sanitary pads and panties ready so they will not be ashamed in class by accidental bleeding. Where there is no access to sanitary pads, girls use a sort of fragmented pieces of cloth*.”

(FGD participant schoolgirl from Arsi Negele Woreda)

#### 3.3.1. Gender, Hygiene and Menstrual Hygiene Management

With regards to menstrual hygiene, there are several misconceptions held by many boys that places additional stress and feelings of shame on girls, especially in school settings. Because of a lack of awareness, boys frequently associate menstruation with sexual activity. Many schoolgirls are constantly in a state of fear that they may have blood stains on their clothing which would result in boys taunting and shaming them for menstruating, both at school and in the community. A lack of adequate water and safe toilets in schools makes it even more difficult to manage bleeding and any soiled clothing. FGD participants shared that even when girls take water with them to use the toilet, there is an assumption that the girl is sexually active [outside of marriage].

“*Even in places where there is access to sanitary pads, women face challenges related to lack of a place for disposal of used sanitary pads. Lack of sanitary pads, water, and privacy in hygienic condition is a very difficult issue in our area. We have no toilet*.”

(FGD participant Woman from Adamitulu)

“*No soap or water in our school. No, nothing. We have no privacy in the school, especially in menstrual time. We lack all the necessary sanitary materials. We are always in the tension of whether some form of blood may be found on our cloth or not*.”

(School Girl FGD participant from Adamitulu)

At the household level, women are responsible for the hygiene conditions of family members including children, elderly, and chronically ill persons. Several FGD participants shared that men only contribute financially and even that is only when needed. Unless women are sick or away from home for different reasons, husbands rarely take responsibility in managing the hygiene situation of their household. In most cases, girls are hesitant to tell their fathers when they are menstruating. Instead, they choose to approach their mothers for support.

“*I give guidance for my girl on how to keep her hygiene and about washing her cloth; I don’t want her to do lots of activities until the end of her menstruation period (I advise her to take rest); I buy her sanitary pads/usually washable pad, soap and other necessary materials which are important for her. But if she hides the information, I cannot help her for I don’t know her condition. These days, girls are getting sanitary pads from their schools through females [girls’] clubs*.”

(FGD participant Woman from Adamitulu)

According to FGD with schoolgirls, the study found that girls clubs are providing sanitary pads procured from the contributions of (0.2USD per two weeks) students. Teachers provide the sanitary pads to girls when they are menstruating, however, most of the girls fear requesting the sanitary pads due to a poor level of awareness and knowledge.

“…*the school provides sanitary pads for a minimum cost, but we don’t go there usually due to shame and fearing of discrimination from boy students*.”

(Schoolgirl from Adami tulu)

#### 3.3.2. Social Exclusion and Hygiene

Findings suggest that SEG have worse hygiene outcomes than other groups due to barriers to water access. Travelling to water points is long and strenuous, especially for elderly persons and PLWD. It is additionally challenging for poor households who do not have tools like donkeys or carts to support the transportation of collected water. As a result, it is common for SEG and poor households to resort to using any water source available nearby which is not safe for personal hygiene or drinking. In addition to exclusion from WASH services, PLWD in the study areas are also excluded from other socio-economic services.

“*The most excluded group are PLWD. No one knows where they are and what they are doing. I myself have a disability. We are excluded from water, sanitation and other different social and economic activities. Buildings are inaccessible for us. In our locality, the community sees PLWs as inferior compared to other [able] groups. This is due to low level of awareness of community. They think disability occurs due to curse*.”

(A key informant from Arsi Negele also a representative of PLWD)

#### 3.3.3. Implications of Hygiene Situation

There are various implications associated with a lack of hygiene facilities in the communities, including implications on health, school attendance, and psychosocial wellbeing.

*Implications on health*: Because of a lack of household level access to water for hygiene, women and girls resort to using rivers to clean themselves during menstruation. This exposes them to water-washed diseases which can negatively impact their health outcomes. On the other hand, handwashing after using the toilet is not routinely practiced. Since water is scarce, families opt to conserve water by eliminating household handwashing. This behavior increases vulnerability to water-borne diseases (including increased diarrhea especially for children).

*Implications on school attendance:* Inadequate sanitation facilities and access to water in schools was found to be negatively associated with school attendance and performance. Due to a lack of privacy, girls feel uncomfortable changing their sanitary pads at school. Lack of water in schools also means girls cannot clean themselves if their clothing becomes soiled, which often results in boys teasing them. As a result, girls often choose to avoid school during menses, resulting in repeated absences in an academic year. Because they regularly miss class, assignments, and exams, girls may have relatively low school performance.

“*When I am menstruating, I drop the class because of pain and go back home. When I miss a class, I also miss different assignments and, in such cases, teachers request me to bring acceptable evidence for my absence. I cannot take evidence since I do not go to health facilities for menstruation. Teachers do not trust me that I missed class due to illness. This affects my school performance*.”

(Schoolgirl FGD participant from Shashemene)

*Psychosocial implications:* Water shortages and inadequate access to water and sanitation facilities often causes women and girls to engage in poor hygiene behavior. Often times, women and girls are criticized and scolded for poor hygiene without consideration of the contributing factors. There are also societal beliefs in the study areas that associate menstruation with sexual activity. These misconceptions drive boys to mistreat and shame girls when they are menstruating. Similarly, the study found that girls and women who are menstruating are often insulted by their peers and sometimes even by their husbands.

## 4. Discussion

Overall, the study found that the study areas are hard hit by water shortage and poor sanitation and hygiene facilities. Fifty-two percent of households included in the study have a basic service level of water, 29% and 14% have basic sanitation and hygiene service levels, respectively [1,3,40]. Lack of adequate sanitation facilities and water lead many to continue to practice open defecation and lack of water was found to be an immediate cause of the sanitation and hygiene problems (diarrheal disease, parasitic infection) experienced by the communities [8,11,12,43,44]. As a cultural norm, the household level roles and responsibilities of water collection and its management are heavily loaded on women and girls, and they carry a disproportionate burden of the challenges associated with lack of water [5,6,10,12,20].

The burden of water management has many implications on the day-to-day lives of women and girls. They usually carry the responsibility of fetching water, travelling long distances, sometimes even into the night, to collect water for their household. This exposes them to safety risks, gender-based violence (e.g., sexual assault), and also health risks (water-borne and water-washed diseases). Women and girls spend a significant portion of their time engaged in household activities, which limits their ability to engage in income generating activities. This often creates a power imbalance in their households and limits their control over resources. Because they spend so much time away from home to secure safe water, their children are sometimes left unattended [7,16,17,18]. For socially excluded groups such as PLWD, chronically ill, and the elderly, the problem of access to safe drinking water has led them to resort to using unprotected and unsafe water sources [12,21,33].

Additionally, the lack of appropriate sanitation facilities had multiple implications for women, girls, and SEG [11,12,24,45]. In areas where open defecation is widely practiced, women fear openly defecating during the daytime due to privacy concerns. They often resort to waiting until after dark to relieve themselves, usually drinking and eating less during the day [16,17,22]. Even during the night, open defecation itself is difficult for women and exposes them to risks of violence [16,17,19]. In schools, lack of WASH facilities, sanitary pads, and space to timely change their pads forces girls to interrupt classes, fall behind on assignments, and often leave school early, which subsequently affects their school attendance and performance [25,26,27,45,46,47,48,49]. Similarly, other SEG also lack access to inclusive (accessible) toilet facilities which forces them to travel to far places to look for open space [12,30]. Women, girls, and other SEG are overall disproportionately affected by inadequate sanitation facilities and shortages of water. Consequently, the health, psychosocial wellbeing, and school performance implications of poor hygiene conditions for schoolgirls and SEGs are significant [24,25,26]. Limited access to water often deters many from routine handwashing practices, putting their health at risk. Resulting unhygienic conditions, due to a lack of water and sanitation facilities, causes women and girls to face criticism and mistreatment by their peers and husbands [23,45,47,48]. In schools, their attendance and ultimately their performance is negatively affected during menstruation because of a lack of sanitary pads, lack of rooms to change their pads and keep their privacy, and misconceptions of boys which results in teasing and shaming [23,24,33,34].

Overall, the absence of adequate and accessible WASH facilities had significant negative affects on SEG, including the elderly and PLWD. There were either no water points that can be easily accessed by SEG due to design flaws, or the water sources were too far for them to travel to. As a result, they regularly resort to collecting water from closer, unsafe water sources [32,35,36,50]. Similarly, adequate latrine access was limited for many SEG. Even where there are toilets, they are not PLWD friendly, forcing these groups to resort to open defecation. At the school level, the toilets were found to be not friendly for both able-bodied children and those with physical disabilities, as the squat holes are too wide. This forces children to defecate just outside the toilets or around the squat holes, which makes the toilets unsanitary for all [45,48].

### Limitation of the Study

Finding reported in this research did not include the perception and awareness of teachers and boys about MHM and the associations with economic status of study areas.

## 5. Conclusions

In the study areas, communities face extreme water shortage and poor sanitation and hygiene facilities. Women and girls are disproportionately affected, as they are the primary care takers of household level water management. The study areas were found to have 52%, 29%, and 14% of water, sanitation and hygiene coverage, and open defecation remains widely practiced. Women are responsible for collecting, handling, storing and treating water for their households. In some cases, men/husbands do not use water economically, despite the limited availability and the difficulty of collection and transportation of water for many women.

Because women are responsible for household level water management, they have limited time to allocate towards income-generating activities. Their responsibility for water activities often exposes them to gender-based violence, leaves them with little time for home management, and results in low education performance for schoolgirls who also contribute to fetching water. Other SEG, on the other hand, resort to collecting water from unprotected or unsafe water sources because they are not able to travel long distances. The design of water points is not inclusive, as WASH development programs do not adequately include the needs of SEG into their design and implementation. This is likely due to a poor level of consultation of the community, including women, during the design, site selection, and construction of water points. Not only should programs work more closely with women, but also with schools and school level girls’ clubs, engaging them in planning. Improving WASH access in schools and promoting better awareness and action around ensuring privacy and access to MHM tools can also help to improve the attendance and performance of schoolgirls.

Social inclusiveness during the design and construction of latrines was also found to be very poor in the study area. It is important that moving forward, communal latrines are designed in a way that is inclusive to PLWD, the elderly, women, and children. Once thorough and participatory consultation and planning is achieved, implementation can be led by the local government, other implementing partners, and the villagers themselves. If communal latrines are inclusive and designed with the community, proper management and timely maintenance of WASH facilities can also be better achieved. To address the issue of women, girls, and SEG, sector offices can coordinate activities using a GESI sensitive WASH budget from the beginning, raising the awareness of communities and ensuring their contribution at all stages of WASH-related development activities.

Training HEWs to apply a GESI lens so they can reach the community through awareness-raising activities in a way that is gender transformative and inclusive could be an important approach to addressing inequality. It is not only the awareness of the general community or government officials which should be prioritized, but rather the awareness and perception of SEG as well. This can help to empower them by building their self-efficacy to demand their WASH service needs are met and that they are represented. Similarly, it is important that the needs of women are represented and met. Men should be willing to do more, in order to reduce the burden of WASH related labor that women face. This can only be achieved by engaging boys and men in community dialogues both at the household and at the community level. Community dialogues can help catalyze shifts in power, so that SEG have increased and equitable decision-making power (‘climb’ on the participation ladder). This requires a ‘top-down’ (policy and practice) approach, but also a bottom-up approach by empowering SEG and men and boys in the community to facilitate changes in attitude and behavior. One of the major gaps to addressing the WASH-related problems of SEG is that there is poor attention given to them by local actors, also reflected by the absence of local supportive policies, strategies, and programs. Findings reported in the study provide vital evidence to inform policy and decision makers to respond in a way that promotes gender equality and inclusive WASH services, in alignment with the SDG targets to be achieved by 2030. It is critical that the inequitable and gendered division of WASH related labor is addressed through government and local actors’ advocacy campaigns. There is a need to work on more awareness activities to make sure that WASH issues impacting SEG are given the necessary attention at all levels of the WASH sector. It is critical that the government contributes to stronger legal practices and policies in order to empower girls, women, and other SEG.

## Data Availability

The data presented in this study are available based on request from the corresponding author. The data are not publicly available due to privacy reasons.

## Appendix A

**Table A1:** Demographic background of focus group discussion participants by Woreda, December 2019.

Name of Woreda	No.	Group Type	Focus Group Discussion Participants
**Shashemene**	1	Elderly Men and Women	9
	2	School Girls (13–19 years)	10
**Arsi Negele**	3	PLWD (both sex)	8
	4	Men	13
	5	Schoolgirls (14–17 years)	11
**Adamitulu Jido Kombolcha**	6	Women	12
	7	Elderly men and women	12
	8	Schoolgirls (13–17 years)	12

**Table A2:** Demographic background of key informants’ participants by Woreda, December 2019.

Key Informant Interview Participants	Study Area		
	Arsi Negele Woreda	Adamitulu Jido Kombolcha Woreda	Shashemene Woreda
Woreda Administrators	1	1	1
Health Office	1	1	
Women and Children/Youth Office	1	1	1
Social Affairs Office	1		1
Water Office	1	1	1
Education Office		1	1
Health Extension Workers	1	1	1
Representatives of PLWD (*Cheshire Ethiopia*)	1		1
Representatives PLHIV		1	
Total KII	**7**	**7**	**7**
